# A New Highly Sensitive Electrochemical Biosensor for Ethanol Detection Based on Gold Nanoparticles/Reduced Graphene Oxide/Polyallylamine Hydrochloride Nanocomposite

**DOI:** 10.3390/bios13110954

**Published:** 2023-10-25

**Authors:** Oana-Maria Istrate, Camelia Bala, Lucian Rotariu

**Affiliations:** 1 LaborQ, University of Bucharest, 030018 Bucharest, Romania; oana-maria.dumitrascu@cdi.unibuc.ro (O.-M.I.); camelia.bala@chimie.unibuc.ro (C.B.); 2 Department of Analytical Chemistry and Physical Chemistry, University of Bucharest, 030018 Bucharest, Romania

**Keywords:** biosensor, ethanol, alcohol dehydrogenase, NADH, gold nanoparticles, reduced graphene oxide, poly (allylamine hydrochloride), alcoholic beverages

## Abstract

A highly sensitive electrochemical biosensor for ethanol based on a screen-printed electrode modified with gold nanoparticles—electrochemically reduced graphene oxide—poly (allylamine hydrochloride) nanocomposite (AuNPs-ERGO-PAH) is reported in this work. Ethanol was oxidized in the presence of the oxidized form of the nicotinamide adenine dinucleotide (NAD^+^) in a reaction catalyzed by alcohol dehydrogenase (ADH) immobilized in sol-gel. The AuNPs-ERGO-PAH nanocomposite was used as a transducer for the electrocatalytic oxidation of the reduced form the nicotinamide adenine dinucleotide (NADH) produced in the enzyme reaction. Under the optimal conditions, the ethanol biosensor exhibits a wide dynamic range from 0.05 to 5 mM with a low detection limit of 10 µM (S/N = 3) and a high sensitivity of 44.6 ± 0.07 µA/mM·cm^2^ for the linear range between 0.05 and 0.2 mM. The biosensor response was stable for up to 6 weeks. Furthermore, the developed biosensor has been used to detect ethanol in alcoholic beverages with good results, suggesting its potential application in various fields, including fermentation processes and food quality control.

## 1. Introduction

Biosensors, especially electrochemical ones, have gained popularity in recent decades for their ability to detect analytes rapidly, with high sensitivity and low detection limits. The most commonly used bioreceptors for biosensing are oxidoreductases, which can be immobilized on electrode surfaces using various physical and chemical methods. Among them, dehydrogenases that use nicotinamide adenine dinucleotide (NAD^+^) or nicotinamide adenine dinucleotide phosphate (NADP^+^) as electron acceptors have significant advantages over oxidases. One of the main advantages is that the reaction catalyzed by dehydrogenases is not affected by oxygen saturation. Additionally, dehydrogenases tend to be more selective than oxidases, which makes them ideal for practical applications. As a result, there has been a surge in published works based on dehydrogenase biosensors in recent years. The analyte detection in a dehydrogenase-based biosensor is achieved by electrochemical detection of the reduced form the nicotinamide adenine dinucleotide (NADH) produced in the oxidation of the analyte in

(1) $$substrate+{NAD}^{+}\;\to \;product+NADH,\;H^{+}$$

the enzyme-catalyzed reaction, which is schematically represented below:

NAD^+^ acts as an acceptor of electrons and hydrogen in this reaction, and the reduced form of the coenzyme is detected using different types of electrodes.

Because of the high overpotential required to oxidize NADH on bare electrodes, the potential side reactions such as dimerization of the coenzyme that can occur and potential electrochemical interferences from other compounds, chemically modified electrodes represent an alternative that can overcome these issues. [1].

A large number of inorganic/organic compounds are used as mediators for the electrochemical detection of NADH such as Prussian blue [2], pyrocatechol violet [3], polineutral red [4], Meldola blue [5,6], nitrocoumarins [7], tetracyanoquinodiemethane (TCNQ), tetrathiafulvalene (TTF) [8], phenothiazine and its derivatives [9].

Carbon nanomaterials and nanocomposites based on carbon nanotubes [3,5,10,11,12] or graphene [13,14,15,16,17] have received increased attention in recent years and proved very efficient for detecting NADH. Mediators can catalyze the electrochemical oxidation of coenzymes at a higher efficiency in the presence of nanomaterials [3,5,6,18,19]. Conductive polymers or polyelectrolytes are also very effective for preparing composite materials with improved properties in detecting NADH at lower potential and high reaction rates [20,21,22,23,24,25]. The integration of gold nanoparticles [25,26,27,28,29] or ionic liquids [30,31] was also successfully realized to develop composites used as electrode materials in detecting NADH.

These types of NADH sensors based on modified electrodes in combination with relevant dehydrogenases can be applied for the preparation of very efficient electrochemical biosensors for the detection of various analytes [32]. Many papers are focused on alcohol detection based on the use of alcohol dehydrogenase (ADH) immobilized on different electrochemical sensors [27,31,33,34,35,36,37,38].

In this work, we report a new ethanol biosensor based on ADH immobilized using the sol-gel technique on the surface of a screen-printed electrode (SPE) modified with a nanocomposite based on gold nanoparticles, reduced graphene oxide and poly (allylamine hydrochloride) (AuNPs-ERGO-PAH) that was developed by our group [39]

**Scheme 1:**
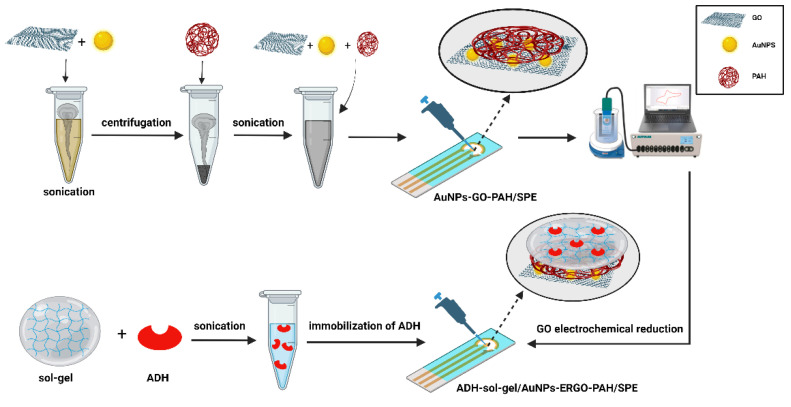
Alcohol dehydrogenase (ADH) biosensor based on a screen-printed electrode (SPE) modified with a nanocomposite based on gold nanoparticles, reduced graphene oxide and poly (allylamine hydrochloride) (AuNPs-ERGO-PAH/SPE) for detection of ethanol. Created with BioRender.com.

The working principle of this biosensor is schematically presented in Scheme 1.

The operational parameters of this biosensor were evaluated and optimized, and its applicability in the detection of ethanol in alcoholic beverages was successfully demonstrated.

## 2. Materials and Methods

### 2.1. Reagents

Nicotinamide adenine dinucleotide reduced disodium salt (NADH), nicotinamide adenine dinucleotide hydrate (NAD^+^), poly (allylamine hydrochloride) (PAH, MW 15 kDa), gold nanoparticles (AuNPs), alcohol dehydrogenase from baker’s yeast (ADH, 75,000 International Units (IU)/g solid), ethanol, ascorbic acid (AA), uric acid (UA), glucose (Glu), methanol (MeOH), were purchased from Sigma Aldrich (Sigma Aldrich Chemie GmbH, Steinheim, Germany) and were used as received without further purification. Graphene oxide (GO) was purchased from Dropsens (Methrom Dropsens, Oviedo, Spain). Tetramethoxysilane (TMOS), methyltrimethoxysilane (MTMOS), and polyethylene glycol (PEG 600) were purchased from Fluka (Fluka Chemie GmbH, Bucks, Switzerland). All other chemicals were of analytical grade. Phosphate buffer (PBS) 0.1 M, pH 8.8, was prepared from Na_2_HPO_4_ and NaH_2_PO_4_ and contains 0.1 M KCl. All solutions were prepared in ultrapure water (Millipore 18 MΩ∙cm).

### 2.2. Equipment and Materials

Cyclic voltammetry and amperometric measurements were performed using an Autolab PGSTAT 101 (Metrohm-Autolab B.V., Utrecht, The Netherlands) Screen-printed carbon electrodes (SPEs) from Dropsens (DRP-C110) (Metrohm Dropsens, Oviedo, Spain) based on carbon working electrode; silver pseudo-reference electrode and carbon counter electrode electrochemical workstation were used for the preparation of the ethanol biosensor. All potentials are reported vs. pseudo-reference silver electrodes. On all the graphs where Δi was shown on the y-axis, the currents were represented versus the baseline recorded in the buffer solution. All measurements were performed at room temperature. The convective transport at constant speed was provided using a magnetic stirrer during the amperometric measurements. An Elmasonic X-tra 50H ultrasonic bath (Elma Schmidbauer GmbH, Singen, Germany) and Qualitron DW 41-230 centrifuge (Qualitron Inc., Gyeonggi-Do, Republic of Korea) were used for the preparation of AuNPs-GO-PAH suspension. A pH meter in the inolab WTW pH 730 (Inolab WTW, Weilhein, Germany) was used to adjust the pH of the buffer solutions. The enzyme activity was determined spectrometrically using a Shimadzu UV-1650PC UV-VIS spectrophotometer (Shimadzu, Kyoto, Japan) by monitoring the NADH at 340 nm. Scanning electron microscopy (SEM) images were recorded with a Carl Zeiss AURIGA CrossBeam Workstation at an accelerating voltage of 2 kV.

### 2.3. Preparation of ADH-Sol-Gel/AuNPs-ERGO-PAH/SPE Biosensor

The first step consisted of the preparation of the ternary composite AuNPs-GO-PAH, which was performed in three stages. The AuNPs-GO composite was obtained by sonication of 1 mL suspension of AuNPs with 25 µL of aqueous suspension of GO (1 mg/mL) for one hour, followed by centrifugation to remove the supernatant. Finally, the third stage was the sonication of 80 µg of AuNPs-GO composite with 40 µL of aqueous PAH solution (1 mg/mL) for 15 min. A total of 5 µL of this composite was deposited on the surface of the carbon working electrode and left at room temperature for 24 h to dry.

The SPE electrode modified with AuNPs-ERGO-PAH composite was prepared by electrochemical reduction of the graphene oxide at the sensor surface using cyclic voltammetry on a potential range between −1000 and +500 mV in KCl solution 0.1 M, in the absence of oxygen for 10 cycles at a scan rate of 10 mV/s. The entire protocol for the preparation of the AuNPs-ERGO-PAH/SPE sensor is described in the previous work published by our research group [39]. AuNPs-ERGO-PAH modified SPE sensor was previously optimized, and the reported results showed an enhanced electrochemical detection of NADH [39].

Before the immobilization, the enzyme activity of ADH was spectrometrically determined by monitoring NADH produced in the enzyme reaction at 340 nm [40]. The kinetic measurements allowed us to determine the maximum reaction rate and express the real enzyme activity.

An ethanol biosensor was then constructed by immobilizing ADH on the AuNPs-ERGO-PAH/SPE surface using the sol-gel matrix. This matrix was prepared with 5 µL of TMOS mixed with 15 µL MTMOS, 40 µL HCl (20 mM), 44 µL ultrapure water, and 4 µL PEG 600. The mixture was sonicated for about 15 min and then kept at 4 °C for 6 h. After hydrolysis of the precursors, 10 µL of the prepared sol-gel was mixed with 10 µL of ADH (20 IU). Finally, 5 IU of ADH were immobilized on the NADH sensor by dropping 5 µL of the mixture on the surface of the modified working electrode. Subsequently, the biosensor was left to dry in a desiccator at 4 °C for 24 h.

## 3. Results and Discussion

### 3.1. Morphological Characterization of the Electrodes

**Figure 1:**
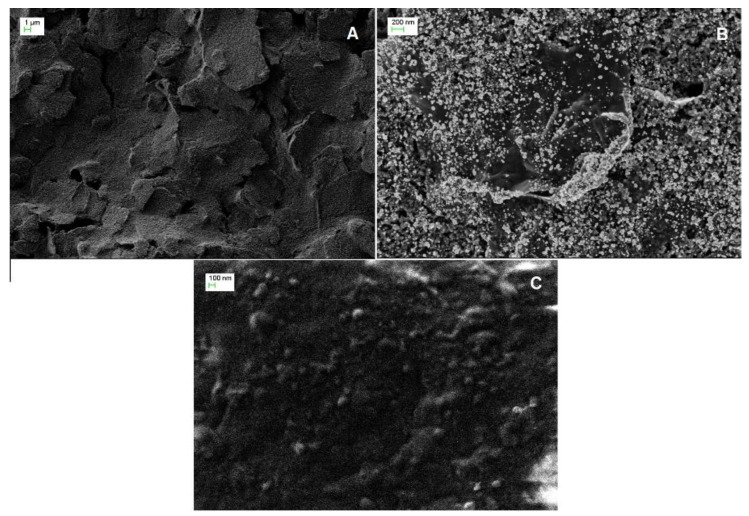
Scanning electron microscopy images of (**A**) SPE, (**B**) AuNPs-ERGO-PAH/SPE, and (**C**) ADH-sol-gel/AuNPs-ERGO-PAH/SPE sensors.

The surface morphology of SPE, AuNPs-ERGO-PAH/SPE, and ADH-sol-gel/AuNPs-ERGO-PAH/SPE electrodes was studied by scanning electron microscopy (SEM). The SEM micrograph of the bare SPE electrode (Figure 1A) shows irregular flakes of graphite, which are randomly oriented. The morphology of the AuNPs-ERGO-PAH composite deposited onto the SPE electrode (Figure 1B) shows the AuNPs included in the composite structure, similar to gelation due to the presence of PAH. A smoother surface was observed after the immobilization of the enzyme with small particles ascribed to the formation of sol-gel in the condensation process in the presence of ADH (Figure 1C).

### 3.2. Cyclic Voltammetry Studies

**Figure 2:**
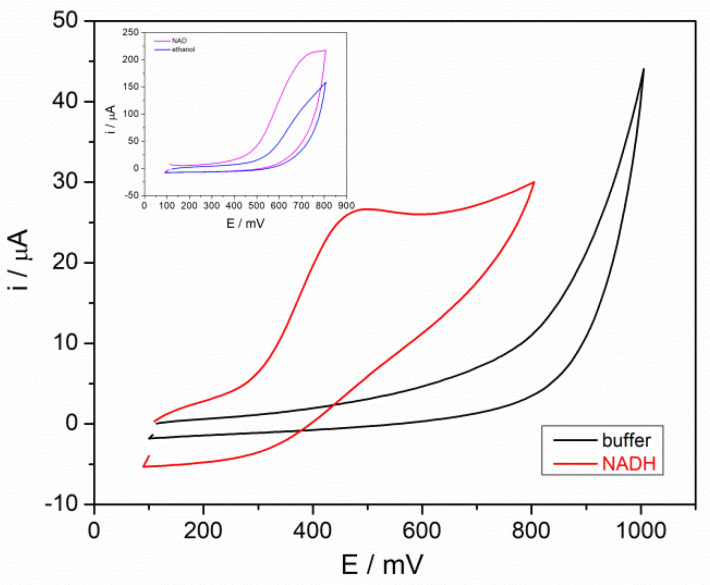
Cyclic voltammograms recorded with ADH-sol-gel/AuNPs-ERGO-PAH/SPE in black line 0.1 M phosphate buffer (PBS), red line 5 mM NADH, pH = 8.8, SR = 100 mV/s, (inset: blue line 3 mM ethanol, pink line 4 mM NAD^+^).

The ADH-based biosensor was tested for detecting NADH, the electrochemical active compound produced in the enzyme reaction catalyzed by ADH, and for detecting ethanol and the oxidized form of the coenzyme (NAD^+^). Cyclic voltammetry studies were performed with the ADH-sol-gel/AuNPs-ERGO-PAH/SPE biosensor in the presence of buffer, 5 mM NADH, 4 mM NAD^+,^ and 3 mM ethanol, respectively. The voltammograms were recorded over a potential range of +100–+1000 mV at a scan rate of +100 mV/s. As shown in Figure 2, the oxidation peak of NADH was recorded at a potential of +480 mV. No oxidation peak for NAD^+^ and ethanol was observed at this potential. However, at a working pH of 8.8, both ethanol and NAD+ carry negative charges and tend to accumulate at the electrode surface due to electrostatic interaction with the PAH-based composite. The increase in current beyond +600 mV could be attributed to this accumulation and may indicate the oxidation of ethanol in the latter case. Therefore, ethanol can be determined by its enzyme oxidation in the presence of NAD^+^, and the electrochemical detection of the NADH produced in this reaction can be correlated with the ethanol concentration.

### 3.3. Optimization of Working Potential

**Figure 3:**
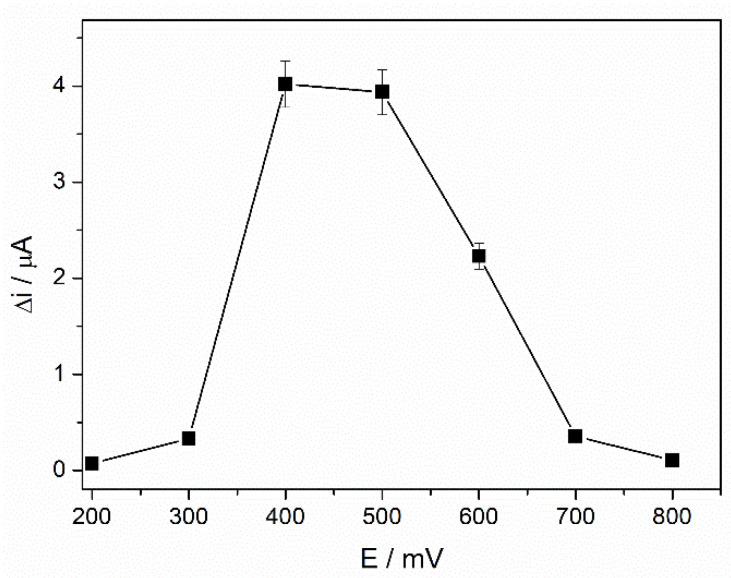
Influence of the working potential on the ADH-sol-gel/AuNPs-ERGO-PAH/SPE response (4 mM NAD^+^, 3 mM ethanol, PBS 0.1 M, pH = 8.8, *n* = 3).

The performances of the amperometric ADH biosensor are affected by the working potential used for detecting the NADH, and optimization of the applied potential can be considered mandatory. Chronoamperometric measurements were performed at different applied potentials in the range +200–+800 mV in 3 mM ethanol and 4 mM NAD+ to achieve this goal. The steady-state signal was recorded before and after each ethanol addition, and the oxidation current variation was determined accordingly. Figure 3 shows how the applied potential affects the electrochemical response of the biosensor. A significant increase in the signal was observed over the potential range between +200 mV and +400 mV, the maximum current being reached at +400 mV. No effective modification was observed at +500 mV, but a significant drop in the current was recorded for higher potentials.

A working potential of +400 mV was considered optimal and used for the following studies. Compared with the ethanol biosensor based on PAH/SPE electrode [41], which requires a working potential of +500 mV for ethanol detection, a lower potential was achieved in the case of the biosensor reported in this work.

### 3.4. Optimization of the Amount of ADH

**Figure 4:**
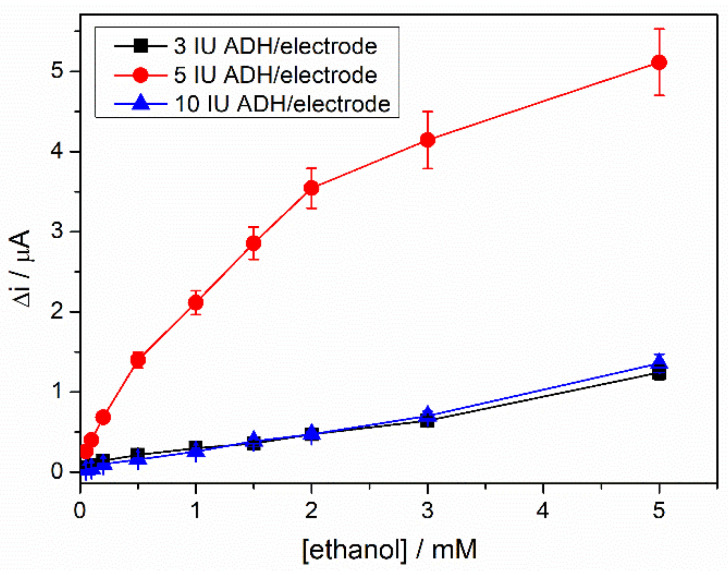
Calibration graphs of ADH-sol-gel/AuNPs-ERGO-PAH/SPE biosensors with 3 International Units (IU) (black line), 5 IU (red line), 10 IU (blue line) ADH (E = +400 mV, 4 mM NAD^+^, 0.1 M PBS, pH = 8.8, *n* = 3).

The amount of enzyme immobilized on the electrode surface is critical for the performance of the biosensor. Usually, a higher amount of ADH leads to an increase in the analytical signal. Still, if the enzyme loading is too high, it can block the electrode surface and consequently lead to a decrease in the biosensor response. In this regard, three different ethanol biosensors were prepared where 3, 5, and 10 IU of ADH were deposited on the surface of the electrode. Chronoamperometric measurements were performed with these biosensors at an applied potential of +400 mV, using an NAD^+^ concentration of 4 mM in 0.1 M PBS, pH = 8.8. Ethanol concentrations were varied between 0.05 and 5 mM. Figure 4 shows the calibration graphs of the three biosensors obtained by the successive addition of ethanol. The biosensor response was significantly enhanced by increasing the amount of enzyme from 3 to 5 IU. A higher amount of enzyme has the opposite effect, leading to a signal drop. Practically, the biosensors with 3 and 10 IU of ADH present almost the same response in the all-ethanol concentration ranges, proving the electrode surface’s blocking effect by the high amount of protein immobilized on it. The highest signal was recorded with the biosensor prepared with 5 IU ADH, which is considered the optimal amount of enzyme for subsequent measurements.

### 3.5. Optimization of the NAD^+^ Concentration

**Figure 5:**
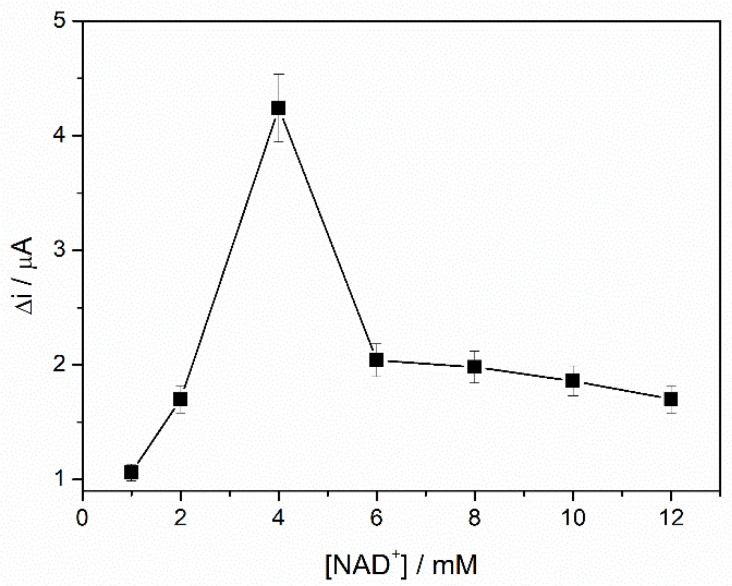
Influence of coenzyme concentration on the ADH-sol-gel/AuNPs-ERGO-PAH/SPE response (E = +400 mV, PBS 0.1 M, pH = 8.8, SR = +100 mv/s, *n* = 3).

The ADH catalyzes the oxidation of the ethanol and requires the presence of NAD^+^, which is reduced to NADH that is electrochemically detected. An excess of NAD^+^ ensures a high reaction rate, consequently leading to a high biosensor response. To study the influence of NAD^+^ concentration on the ADH-sol-gel/AuNPs-ERGO-PAH/SPE biosensor response, chronoamperometric measurements were performed at an applied potential of +500 mV for an ethanol concentration of 3 mM, while the NAD^+^ concentration was varied in the range of 1 to 12 mM. The biosensor’s analytical signal, depicted in Figure 5, increases in the concentration range between 1 and 4 mM NAD^+,^ reaching a maximum of 4 mM. A significantly lower amount of NAD^+^ is required in the case of this biosensor compared to the ADH-sol-gel/PAH/SPE biosensor previously reported [41], for which the optimal concentration was 10 mM. The presence of nanocomposite based on AuNPs and RGO that accelerated the transfer of electrons at the biosensor surface can explain this behavior. A decrease of the oxidation current was observed at higher concentrations of NAD^+^, probably due to a blocking of the biosensor surface by accumulating an excess of NAD^+^.

The concentration of 4 mM of NAD^+^ was considered optimal, and it was used for the calibration of the biosensor and the detection of ethanol in the real samples.

### 3.6. Optimization of the Working pH

**Figure 6:**
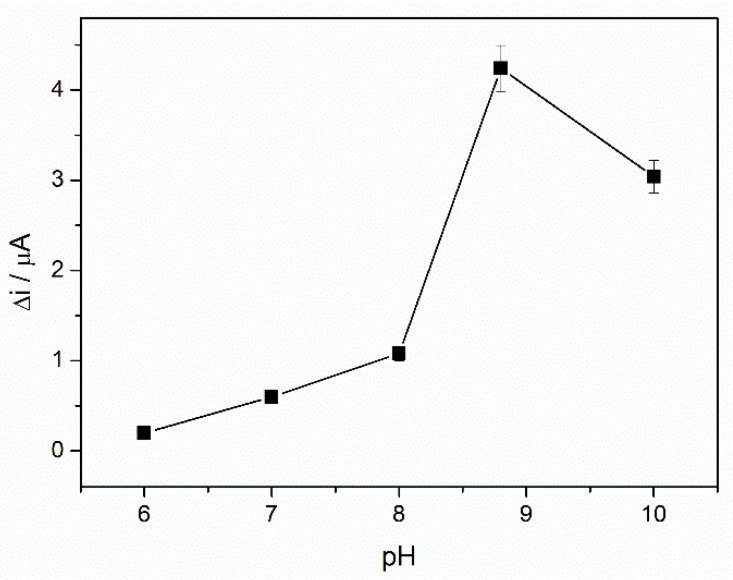
Influence of working pH on the ADH-sol-gel/AuNPs-ERGO-PAH/SPE response (4 mM NAD^+^, 3 mM ethanol, PBS 0.1 M, E = +400 mV, *n* = 3).

The pH is an important parameter for all enzyme-based biosensors related to the enzyme reaction rate and the electrostatic interaction between substrates and the electrode surface. To optimize the working pH of the ADH-sol-gel/AuNPs-ERGO-PAH/SPE biosensor, chronoamperometric measurements were performed at an applied potential of +400 mV in the presence of 4 mM NAD^+^ and 3 mM ethanol, varying the buffer pH in the range from 6 to 10. The buffer with pH 10 was prepared by adjusting the pH of the PBS buffer (pH 8.8) with NaOH 0.1 M. As can be seen from Figure 6, the analytical signal increases over the pH range from 6 to 8, reaching a maximum pH value of 8.8. The optimal pH of the ADH reported in the literature is between 8 and 9. Therefore, an optimal pH of 8.8, observed for the biosensor reported in this work, shows that the immobilized enzyme acts as the native enzyme. Moreover, this pH favors the accumulation of NAD^+^ at the surface of the composite-modified electrode. Consequently, all subsequent studies were performed at pH 8.8.

### 3.7. Calibration Curve of ADH-Sol-Gel/AuNPs-ERGO-PAH/SPE Biosensor

The calibration of ADH-sol-gel/AuNPs-ERGO-PAH/SPE biosensor was performed by amperometry in a 5 mL electrochemical cell provided with a magnetic stirrer at an applied potential of +400 mV in the presence of 4 mM NAD^+^ by successive additions of ethanol. The baseline current was used as a reference for all signals recorded after each ethanol addition.

**Figure 7:**
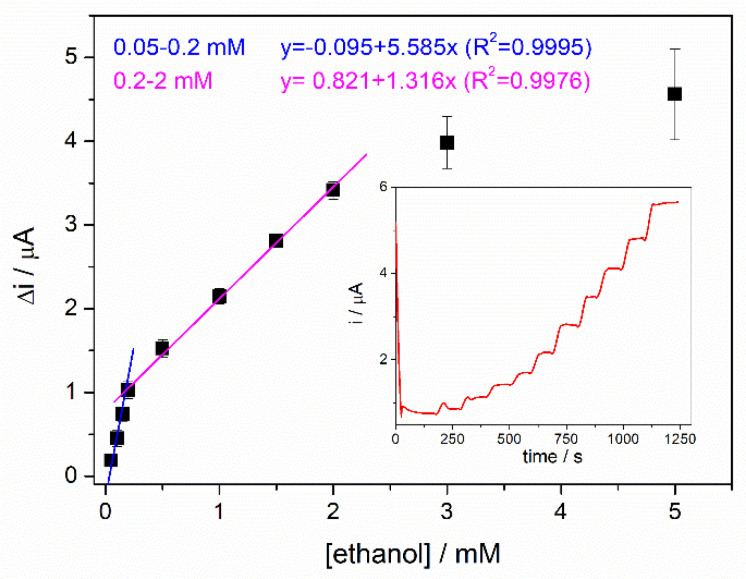
Calibration curve of ADH-sol-gel/AuNPs-ERGO-PAH/SPE biosensor (4 mM NAD^+^, PBS 0.1 M pH = 8.8, E = +400 mV; *n* = 3) (inset: typical current-time response curve for ethanol).

The response curve of the biosensor corresponding to the range of ethanol concentrations between 0.05 and 5 mM is shown in Figure 7, where each point represents the mean value of 3 determinations. Two linear ranges were extracted from this graph. For the first linear range between 0.05 and 0.2 mM, a specific sensitivity of 44.6 ± 0.07 µA/mM·cm^2^ was determined, and a detection limit of 10 µM was calculated for the signal/noise ratio equal to 3. A second linear range was assigned to the domain between 0.2 and 2 mM ethanol with a lower specific sensitivity of 10.51 ± 0.03 µA/mM·cm^2^. The enzyme saturation effect was observed at ethanol concentrations higher than 2 mM. The standard deviation of the measurements in this range is almost the same range as the sensitivity of the potential third linear range that could be described from 2 to 5 mM. Thus, it is difficult to distinguish between concentrations that vary by 1 mM, and this range is not helpful for real sample applications. To cover the broad range of ethanol concentrations in alcoholic beverages, it is highly desirable to have a biosensor with a wider working response range that can accommodate all measurements between 0.02 and 2 mM using a simple sample preparation procedure; this will eliminate the need for multiple dilutions of the same sample to fit into a narrow calibration range. With a unique protocol for sample preparation, accurate results can be obtained, as shown in the proof of concept described in Section 3.10.

The response time of the biosensor is approximately 24 s, calculated as the time required to reach 90% of the steady state signal.

Based on the experimental results, the biosensor developed in this study has proven to be highly effective in detecting ethanol across a broad range of concentrations. The biosensor operates at low potential values, exhibits high sensitivity, and has a low detection limit when compared to the biosensor developed by our research group in the past, which was based on a PAH/SPE electrode [41]. Table 1 compares the performance of different ethanol biosensors based on composite-modified electrodes published in the literature, and it shows that the biosensor developed in this study has one of the highest specific sensitivity levels. The biosensor presented in this study has a high specific sensitivity of 44.6 µA/mM·cm^2^, which is two to ten times higher than other ethanol biosensors listed in Table 1. Although the biosensor developed by Wang et al. [12] showed a slightly higher sensitivity and a lower working potential, its narrow linear range makes it unsuitable for real sample determinations. Another ethanol biosensor based on Au-AgNPs/P(l-Cys)-ERGO/GCE [25] had a working concentration range of approximately two decades based on two linear ranges but had a sensitivity approximately 10 times lower than that of the biosensor developed in this study. While other research groups’ ethanol biosensors [10,42] have a lower detection limit compared to the developed biosensors, they lack a wide response range and high sensitivity. Therefore, we believe that the ethanol biosensor based on AuNPs-ERGO-PAH/SPE electrode exhibits good performance by combining high sensitivity, wide working range, and low detection limit.

### 3.8. Stability of the ADH-Sol-Gel/AuNPs-ERGO-PAH/SPE Biosensor

**Figure 8:**
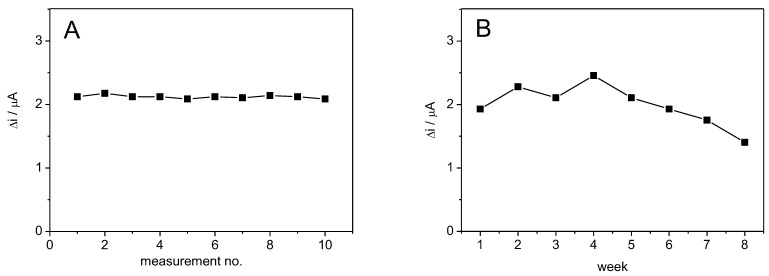
(**A**) Operational stability and (**B**) Long-term stability of ADH-sol-gel/AuNPs-ERGO-PAH/SPE biosensor. (E = +400 mV, [NAD^+^] = 4 mM, 1 mM ethanol, PBS 0.1 M pH = 8.8).

Chronoamperometric measurements were performed to study the operational stability of the ADH-sol-gel/AuNPs-ERGO-PAH/SPE. Figure 8A shows the biosensor response for 10 successive measurements performed every 2 min at an applied potential of +400 mV in 0.1 M PBS buffer containing 4 mM NAD^+^ and 1 mM ethanol. Between measurements, the biosensor was rinsed with buffer 0.1 M PBS. The relative standard deviation (RSD) of 1.45% was calculated. This value indicates good operational stability of the ethanol biosensor proposed in this paper compared to the ethanol biosensor previously reported by our group [41].

Long-term operational stability was studied by chronoamperometric measurements performed once a week for 6 weeks using the same biosensor. Between measurements, the biosensor was kept in the desiccator at 4 °C. Figure 8B shows a stable biosensor response for 5 weeks, followed by a gradual decrease to 70% at 8 weeks.

### 3.9. Selectivity of the ADH-Sol-Gel/AuNPs-ERGO-PAH/SPE Biosensor

The selectivity of the biosensor was studied by chronoamperometric measurements in 0.1 M PBS solution, pH = 8.8 with 4 mM NAD^+^, 1 mM ethanol at an applied potential of +400 mV by successive addition of 1 mM of methanol, glucose, uric acid, and ascorbic acid, respectively. Finally, the biosensor response was recorded after adding 1 mM ethanol.

**Figure 9:**
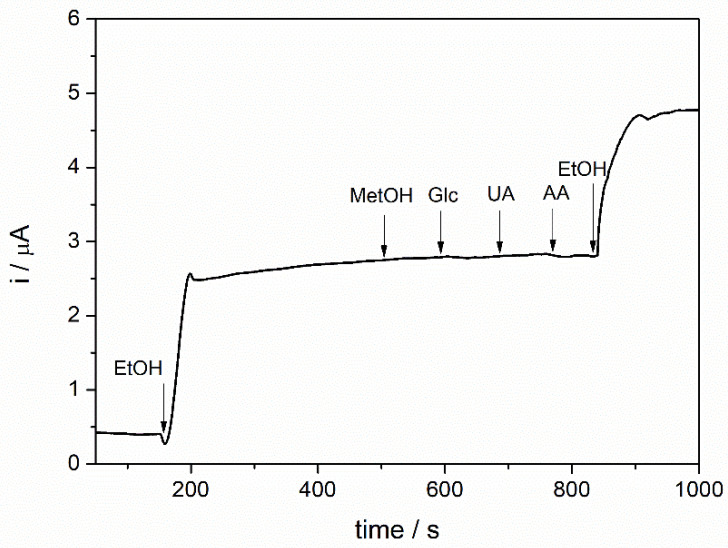
Biosensor response to adding 1 mM ethanol and 1 mM of methanol, glucose, uric acid, and ascorbic acid; E = +400 mV, [NAD+] = 4 mM, PBS 0.1 M pH = 8.8).

The response of the biosensor is shown in Figure 9, where a well-defined response to ethanol is observed, while the four potential interferent compounds showed no visible response. The final addition of ethanol showed a similar oxidation current compared with the first ethanol addition. The results demonstrated that the ADH-sol-gel/AuNPs-ERGO-PAH/SPE biosensor had good selectivity toward the studied interfering compounds, good stability, and reproducibility and can be successfully applied for ethanol detection in real samples.

### 3.10. Analysis of Alcoholic Beverages Samples

In order to demonstrate the practicality of the ethanol biosensor for testing real samples, five different alcoholic beverages were examined, including beer, aperitif drink, sparkling wine, and herbal and lemon liqueur. These drinks were chosen from commercially available products. Before testing, the samples were diluted 1:100 in ultrapure water. The proposed ethanol biosensor, like other dehydrogenase-based biosensors, utilizes NAD+ as a coenzyme. Therefore, a 20 mM NAD+ solution must be prepared before conducting the analysis of real samples. To determine the ethanol content, a 5 mL electrochemical cell was used with the conditions previously optimized in other studies by adding 4.85 mL of PBS, 100 µL of 20 mM NAD+, and 50 µL of the diluted beverage sample; this resulted in a final concentration of 4 mM NAD+ and a second dilution of 1:100 of the beverage sample in the electrochemical cell. The ethanol concentration in the tested drinks was calculated based on the experimental protocol and sample dilution. The first linear range was used for beverages with low ethanol content, while the second was used for liquors. Table 2 shows a comparison between the ethanol content determined experimentally with the developed biosensor and the ethanol content indicated on the product label.

It is possible that the complexity of the beer sample, along with other oxidation-reduction processes, may have contributed to an increase in the biosensor response; this could be the reason for the higher concentration of ethanol found in this particular sample. Another possible explanation could be the high dilution levels. However, the other four alcoholic beverages showed a strong correlation between the detected ethanol concentration and the ethanol content declared by the producer, with recoveries ranging from 97% to 108%. Overall, the experimental results validate that the ethanol biosensor described in this study can be a useful tool for detecting ethanol in commercial alcoholic beverages.

## 4. Conclusions

In this study, a new biosensor for the detection of ethanol was developed. It is based on alcohol dehydrogenase immobilized on the surface of an SPE electrode modified with a nanocomposite material. The synergetic effect of polyallylamine hydrochloride, gold nanoparticles, reduced graphene oxide, and the ADH were exploited to detect NADH produced in the enzymatic oxidation of ethanol. Optimization of the main operational parameters such as the applied potential, amount of enzyme, amount of coenzyme, or pH were performed. The biosensor exhibited good sensitivity, a low detection limit, good reproducibility, and stability for up to 6 weeks. The simple manufacturing method with low costs and good analytical performances recommends the ADH-sol-gel/AuNPs-ERGO-PAH/SPE biosensor for application in detecting ethanol from various samples, including alcoholic beverages.

## Figures and Tables

**Table 1:** Comparison of the performances of some ethanol biosensors based on polymers and nanomaterials.

Electrode Material	E (mV)	Linear Range (µM)	LOD (µM)	Sensitivity (µA/mM·cm^2^)	Ref.
AuNPs/NFPBA/SPBM	+600	500–50,000	N.R.	0.7	[38]
AH/IH/MWCNTs/GCE	+150	20–400	5	68.53	[12]
TCBQ-LCPs/SWCNTS/GCE	+130	200–13,000	50	3.65	[36]
Au-AgNPs/P(l-Cys)-ERGO/GCE	+350	17–658 658–1845	5	4.549 1.25	[25]
SWCNTs-rGO/GCE	+500	5–400	0.16	26.27	[42]
MWCNTs-Polyarg/GCE	+350	5–100	0.65	4.461	[10]
PBNPs-CMC/SPE	+400	0–1000	300	-	[43]
PAH/SPE	+500	50–200 200–2000	20	13.45 5.74	[41]
AuNPs-ERGO-PAH/SPE	+400	50–200 200–2000	10	44.6 10.51	This work

AuNPs-nano cubiAuNPs–gold nanoparticles, NFPBA-Nickel hexacyanoferrate, SPBM–screen-printed biosensing microchips, AH–aluminum hydroxide, IH–iron hydroxide, MWCNTs–multiwalled carbon nanotubes, TCBQ–chloranil (2,3,5,6-tetrachloro-1,4-benzoquinone), LCPs–liquid-crystalline cubic phases, Au-AgNPs–gold-silver bimetallic nanoparticles, P(l-Cys)–poly (l-cysteine), ERGO-reduced graphene oxide, GCE–glassy carbon electrode, MWCNTs—carbon nanotubes, Polyarg—poly-L-arginine, GCE—glassy carbon electrode, PBNPs—hydrogel films embedding Prussian Blue nanoparticles, CMC—hydrogel scaffold based on carboxymethyl cellulose, SPE—screen printed electrode, PAH–poly (allylamine hydrochloride), SPE–screen printed electrode.

**Table 2:** Determination of ethanol in commercial alcoholic beverages with ADH-sol-gel/AuNPs-ERGO-PAH/SPE.

Sample	Found (M)	Commercial (M)	Recovery (%)
Beer	1.12	0.86	130 ± 12
Aperitif drink	1.95	1.80	108 ± 10
Sparkling wine	1.90	1.88	101.1 ± 9.2
Herbal liqueur	6.25	6.09	103 ± 11
Lemon liqueur	5.02	5.14	97.6 ± 8.5
